# Supplementary material for: Exergames in Older Adult Community Centers and Nursing Homes to Improve Balance and Minimize the Risk of Falls in Older Adults: A Systematic Review and Meta-Analysis
Source: Healthcare (Basel). 2023 Jun 28;11(13):1872. doi: 10.3390/healthcare11131872 (PMC10341223; doi:10.3390/healthcare11131872)
Supplement: Supplementary file 1 [file healthcare-11-01872-s001.zip › Supplementary material 2.pdf]

Supplementary material 2: Organization of searches

(Aged) OR (Frail Elderly) OR (Aged, 80 and over) OR ("Frail Elderly") OR ("Aged, 80 and over") OR (Elderly) OR (Elderly, Frail) OR (Frail Elders) OR (Elder, Frail) OR (Frail Older Adults) OR (Adult, Frail Older) (Frail Older Adult) OR (Oldest Old) AND (Video Games) OR (Exergaming) OR ("Video Games") OR (Game, Video) OR (Games, Video) OR (Video Game) OR (Computer Games) OR (Computer Game) OR (Game, Computer) OR (Games, Computer) (Exergame) OR (Exergames) OR (Exergamings) OR (Active-Video Game) OR (Active Video Gaming) OR (Active-Video Gamings) OR (Gaming, Active-Video) OR (Virtual Reality Exercise) OR (Exercise, Virtual Reality) OR (Virtual Reality Exercises) AND (Accidental Falls) OR (Postural Balance) OR ("Accidental Falls") OR (“Postural Balance”) OR (Falls) OR (Fall) OR (Falling) OR (Falls, Accidental) OR (Accidental Fall) OR (Fall, Accidental) OR (Slip and Fall) OR (Fall and Slip) OR (Balance) OR (Posture Equilibrium) OR (Equilibrium, Posture) OR (Posture Equilibriums) OR (Balance, Postural) OR (Postural Equilibrium) OR (Equilibrium)

Aged OR Frail Elderly OR Aged, 80 and over OR "Frail Elderly" OR "Aged, 80 and over" OR Elderly OR Elderly, Frail OR “Elderly, Frail” OR Frail Elders OR “Frail Elders” OR Elder, Frail OR “Elder, Frail” OR Frail Older Adults OR “Frail Older Adults” OR Adult, Frail Older OR “Adult, Frail Older” OR Frail Older Adult OR “Frail Older Adult” OR Oldest Old OR “Oldest Old” AND Video Games OR Exergaming OR "Video Games" OR Game, Video OR “Game, Video” OR Games, Video OR “Games, Video” OR Video Game OR “Video Game” OR Computer Games OR “Computer Games” OR Computer Game OR “Computer Game” OR Game, Computer OR “Game, Computer” OR Games, Computer OR “Games, Computer” OR Exergame OR Exergames OR Exergamings OR Active-Video Game OR “Active-Video Game” OR Active Video Gaming OR “Active Video Gaming” OR Active-Video Gamings OR “Active-Video Gamings” OR Gaming, Active-Video OR “Gaming, Active-Video” OR Virtual Reality Exercise OR “Virtual Reality Exercise” OR Exercise, Virtual Reality OR “Exercise, Virtual Reality” OR Virtual Reality Exercises OR “Virtual Reality Exercises” AND Accidental Falls OR Postural Balance OR "Accidental Falls" OR “Postural Balance” OR Falls OR Fall OR Falling OR Falls, Accidental OR “Falls, Accidental” OR Accidental Fall OR “Accidental Fall” OR Fall, Accidental OR “Fall, Accidental” OR Slip and Fall OR “Slip and Fall” OR Fall and Slip OR “Fall and Slip” OR Balance OR Posture Equilibrium OR “Posture Equilibrium” OR Equilibrium, Posture OR “Equilibrium, Posture” OR Posture Equilibriums OR “Posture Equilibriums” OR Balance, Postural OR “Balance, Postural” OR Postural Equilibrium OR “Postural Equilibrium” OR Equilibrium
