# Supplementary material for: Exergames in Older Adult Community Centers and Nursing Homes to Improve Balance and Minimize the Risk of Falls in Older Adults: A Systematic Review and Meta-Analysis
Source: Healthcare (Basel). 2023 Jun 28;11(13):1872. doi: 10.3390/healthcare11131872 (PMC10341223; doi:10.3390/healthcare11131872)
Supplement: Supplementary file 1 [file healthcare-11-01872-s001.zip › Supplementary material 3.pdf]

Supplementary material 3: Summary for analyzing the quality of evidence (GRADE)

Certainty assessment							№ of patients		Effect		Certainty	Importance
№ of studies	Study Design	Risk of Bias	Inconsistency	Indirect evidence	Imprecision	Other considerations	Exergame	Control	Relative (95% CI)	Absolute (95% CI)		
**Risk of falls**												
17	Randomized Clinical Trials	Very serious[a]	Seriouse[b]	Serious[c]	Not Serious	strong association all potential confounders would reduce the demonstrated effect	563	559	-	SMD **0.234 SD more hith** (0.111 more hith for 0.357 more hith)	⊕⊕◯◯ Low	CRÍTICAL
**Balance**												
15	Randomized Clinical Trials	Very serious[a]	Serious[d]	Serious[c]	Not Serious	strong association all potential confounders would reduce the demonstrated effect	440	439	-	SMD **0.325 SD more hith** (0.204 more hith for 0.447 more hith)	⊕⊕◯◯ Low	CRÍTICAL

CI: Confidence interval; **SMD:** Standardised mean difference

Explanations

a. In the evaluation of Risk of bias (The Cochrane Collaboration's tool) the items "Blinding of participants and personnel" and "Blinding of outcome assessment" were mostly evaluated for "High risk of bias", due to non-blinding

b. For the risk of falls, there is significant heterogeneity within the groups (P=0.014) and there is no significant heterogeneity between the groups (P=0.939). In the individual verification, it is noted that significant heterogeneity occurs in the group of PPA studies. The others do not have significant heterogeneity. Others: I2 - 13.24% (p 0.31) PPA: I2 - 76.00% (p 0.02) TUG: I2 - 25.71% (p 0.19)

c. The games used are commercial, which were not developed to treat the outcomes studied. There were problems with the interventions, such as not monitoring variables such as intensity. The interventions carried out had a short duration on average and there was no follow-up to verify adherence to treatment after the experiment.

d. Both are statistically significant, as is the summary measure of the subgroups. However, there is still great heterogeneity within the groups (P<0.001) and none between the groups (P=0.93). Both groups have large and significant heterogeneities. BBS: I2 - 61.89% (p 0.003) Others: I2 - 74.05% (p 0.006)
