# Supplementary material for: Exergames in Older Adult Community Centers and Nursing Homes to Improve Balance and Minimize the Risk of Falls in Older Adults: A Systematic Review and Meta-Analysis
Source: Healthcare (Basel). 2023 Jun 28;11(13):1872. doi: 10.3390/healthcare11131872 (PMC10341223; doi:10.3390/healthcare11131872)
Supplement: Supplementary file 1 [file healthcare-11-01872-s001.zip › Supplementary material 5.pdf]

Supplementary material 6: Assessing risk of bias in the non-randomized study

Study	Bias due to confounding	Bias in selection of participants into the study	Bias in classification of interventions	Bias due to deviations from intended interventions	Bias due to missing data	Bias in measurement of outcomes	Bias in selection of the reported result
[1]	Moderate risk of bias	Low risk of bias	Moderate risk of bias	Low risk of bias	Low risk of bias	Low risk of bias	Moderate risk of bias

References:

1. Batista, J.S.; Wibelinger, L.M.; De Marchi, A.C.B.; Pasqualotti, A. Evaluation and Physiotherapeutic Intervention in Older with Deficit Balance through the Scale of Berg and Wii Balance Board Platform. *Fisioter. em Mov.* **2014**, *27*, 21–28, doi:10.1590/0103-5150.027.001.ao02.
2. Sterne, J.A.; Hernán, M.A.; Reeves, B.C.; Savović, J.; Berkman, N.D.; Viswanathan, M.; Henry, D.; Altman, D.G.; Ansari, M.T.; Boutron, I.; et al. ROBINS-I: A Tool for Assessing Risk of Bias in Non-Randomised Studies of Interventions. *BMJ* **2016**, i4919, doi:10.1136/bmj.i4919.
