# Supplementary material for: Exergames in Older Adult Community Centers and Nursing Homes to Improve Balance and Minimize the Risk of Falls in Older Adults: A Systematic Review and Meta-Analysis
Source: Healthcare (Basel). 2023 Jun 28;11(13):1872. doi: 10.3390/healthcare11131872 (PMC10341223; doi:10.3390/healthcare11131872)
Supplement: Supplementary file 1 [file healthcare-11-01872-s001.zip › Supplementary material 6 -.pdf]

Supplementary material 6: Other meta-analysis

6.1 Risk of falls

Figure 1: Results of all measurements – risk of falls studies.

Study name	Outcome	Std diff in means	Lower limit	Upper limit	p-Value	Exergame	Control
		Statistics for each study				Sample size	
Bieryla, Dold, 2014a	Risk of Falls	0,147	-1,094	1,389	0,816	5	5
Bieryla, Dold, 2014b	Risk of Falls	0,042	-1,198	1,282	0,947	5	5
Delbaere et al, 2021a	Risk of Falls	0,081	-0,094	0,256	0,366	254	249
Delbaere et al, 2021b	Risk of Falls	0,011	-0,164	0,186	0,904	254	249
Delbaere et al, 2021c	Risk of Falls	0,125	-0,050	0,300	0,161	254	249
Delbaere et al, 2021d	Risk of Falls	0,250	0,075	0,425	0,005	254	249
Delbaere et al, 2021e	Risk of Falls	0,117	-0,058	0,292	0,189	254	249
Delbaere et al, 2021f	Risk of Falls	0,036	-0,139	0,211	0,684	254	249
Delbaere et al, 2021g	Risk of Falls	0,000	-0,175	0,175	1,000	254	249
Franco et al, 2011	Risk of Falls	-0,047	-0,903	0,810	0,915	11	10
Fu et al (2015)	Risk of Falls	0,815	0,288	1,341	0,002	30	30
Gschwind et al, 2015 KINa	Risk of Falls	0,155	-0,365	0,675	0,560	29	28
Gschwind et al, 2015 KINb	Risk of Falls	0,000	-0,519	0,519	1,000	29	28
Gschwind et al, 2015 SMRa	Risk of Falls	0,056	-0,355	0,467	0,790	47	44
Gschwind et al, 2015 SMRb	Risk of Falls	0,353	-0,061	0,767	0,095	47	44
Hsieh et al, 2014	Risk of Falls	-0,221	-1,611	1,169	0,756	4	4
Lai et al, 2013a	Risk of Falls	0,286	-0,433	1,006	0,435	15	15
Lai et al, 2013b	Risk of Falls	-0,157	-0,874	0,560	0,668	15	15
Aguinaga; Zapirain, 2017	Risk of Falls	1,151	0,473	1,828	0,001	20	19
Phirom et al, 2020a	Risk of Falls	0,457	-0,179	1,093	0,159	19	20
Phirom et al, 2020b	Risk of Falls	1,052	0,382	1,722	0,002	19	20
Phirom et al, 2020c	Risk of Falls	0,848	0,192	1,503	0,011	19	20
Pina et al, 2015	Risk of Falls	0,208	-0,775	1,191	0,678	8	8
Rendon et al, 2012	Risk of Falls	0,021	-0,653	0,694	0,952	16	18
Sato et al, 2015	Risk of Falls	0,270	-0,252	0,791	0,311	29	28
Szturm et al, 2011	Risk of Falls	1,408	0,565	2,252	0,001	13	14
Treml et al, 2013	Risk of Falls	0,112	-0,582	0,805	0,752	16	16
Yang et al, 2020a	Risk of Falls	0,000	-0,877	0,877	1,000	10	10
Yang et al, 2020v	Risk of Falls	0,349	-0,534	1,232	0,439	10	10
Yesilyaprak et al, 2016a	Risk of Falls	0,446	-0,513	1,404	0,362	7	11
Yesilyaprak et al, 2016b	Risk of Falls	0,112	-0,836	1,060	0,817	7	11
Zahedian-Nasab et al, 2021a	Risk of Falls	0,016	-0,490	0,523	0,949	30	30
Zahedian-Nasab et al, 2021b	Risk of Falls	0,513	-0,002	1,027	0,051	30	30

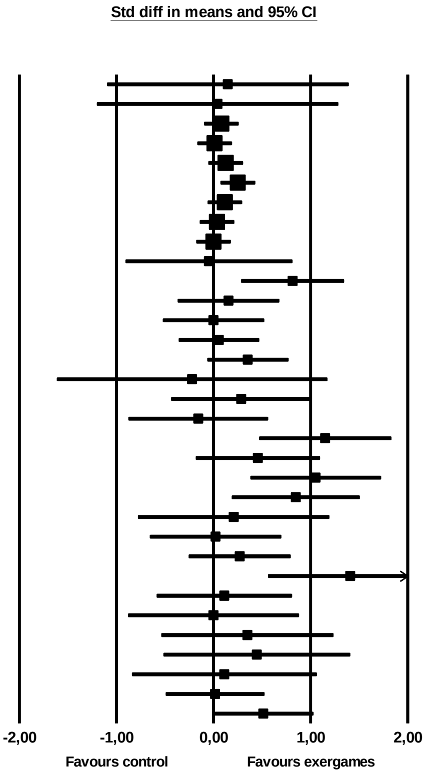

Meta Analysis

This image is important for viewing all studies results and their directions

Figure 2: Subgroup analysis: organization by the control group - with or without intervention -

Group by Control Type	Study name	Outcome	Statistics for each study				Sample size	
			Hedges'g	Lower limit	Upper limit	p-Value	Exergame	Control
With Intervention	Delbaere et al, 2021a	Risk of Falls	0,081	-0,094	0,255	0,366	254	249
With Intervention	Fu et al (2015)	Risk of Falls	0,804	0,284	1,324	0,002	30	30
With Intervention	Gschwind et al, 2015 KIN	Risk of Falls	0,153	-0,360	0,665	0,560	29	28
With Intervention	Gschwind et al, 2015 SM[illegible]	Risk of Falls	0,055	-0,352	0,463	0,790	47	44
With Intervention	Lai et al, 2013a	Risk of Falls	0,279	-0,421	0,979	0,435	15	15
With Intervention	Phirom et al, 2020c	Risk of Falls	0,830	0,188	1,472	0,011	19	20
With Intervention	Pina et al, 2015	Risk of Falls	0,197	-0,732	1,126	0,678	8	8
With Intervention	Szturm et al, 2011	Risk of Falls	1,366	0,548	2,183	0,001	13	14
With Intervention	Treml et al, 2013	Risk of Falls	0,109	-0,567	0,785	0,752	16	16
With Intervention	Yang et al, 2020a	Risk of Falls	0,000	-0,839	0,839	1,000	10	10
With Intervention	Yesilyaprak et al, 2016a	Risk of Falls	0,424	-0,489	1,337	0,362	7	11
With Intervention	Zahedian-Nasab et al, 20[illegible]	Risk of Falls	0,016	-0,483	0,516	0,949	30	30
With Intervention			0,300	0,086	0,514	0,006		
Without Intervention	Bieryla, Dold, 2014a	Risk of Falls	0,133	-0,988	1,254	0,816	5	5
Without Intervention	Franco et al, 2011	Risk of Falls	-0,045	-0,867	0,777	0,915	11	10
Without Intervention	Hsieh et al, 2014	Risk of Falls	-0,192	-1,401	1,017	0,756	4	4
Without Intervention	Aguinaga; Zapirain, 2017	Risk of Falls	1,127	0,463	1,791	0,001	20	19
Without Intervention	Rendon et al, 2012	Risk of Falls	0,020	-0,637	0,678	0,952	16	18
Without Intervention	Sato et al, 2015	Risk of Falls	0,266	-0,249	0,780	0,311	29	28
Without Intervention			0,288	-0,109	0,685	0,156		
Overall			0,297	0,109	0,486	0,002		

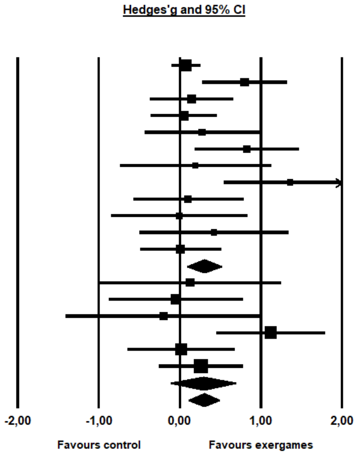

Interventions	I^2	P
With Interventions	45.271%	0.043
Without Interventions	38.004%	0.152

Here the number of studies decreases, as we cannot place the same individuals in the same subgroup. In all cases, the results of the scales most used were maintained.

There was significance only in studies with the control group with intervention. The summary measure, which combines the subgroups, remains significant.

Heterogeneity within subgroups was significant (P = 0.03) and between subgroups there was no heterogeneity (P = 0.49).

Although without significance in the non-intervention group, both groups still had heterogeneities.

Figure 3: Subgroup analysis - duration of intervention

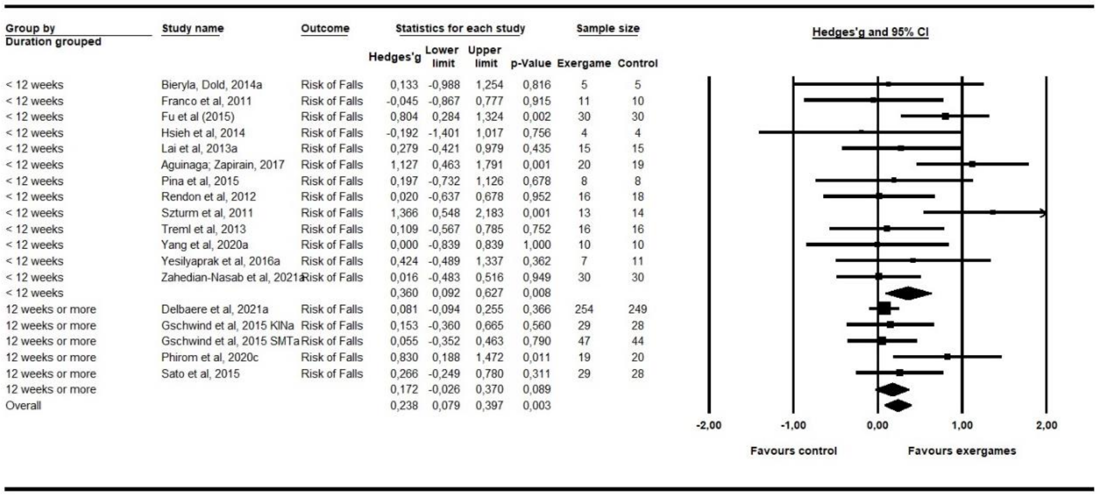

Duration	I2	P
< 12 weeks	39.690%	0.069
12 weeks or more	24.213%	0.259

There was significance only in the < 12 weeks group. The summary measure that combines the subgroups remains significant. There was no significant heterogeneity within the groups (P = 0.067) and between groups (P = 0.062).

In the individual analysis, there were no significant heterogeneities. However, there are still high I^2 values, especially in the < 12 weeks group.

Figure 4: Subgroup analysis – place of execution

Group by Place	Study name	Outcome	Statistics for each study: Hedges's g	Standard error	Lower limit	Upper limit	p-Value	Sample size: Exergame	Control
Community	Bieryla, Dold, 2014b	Risk of Falls	0,038	0,571	-1,082	1,158	0,947	5	5
Community	Delbaere et al, 2021a	Risk of Falls	0,081	0,089	-0,094	0,255	0,366	254	249
Community	Delbaere et al, 2021	Risk of Falls	0,011	0,089	-0,164	0,185	0,904	254	249
Community	Franco et al, 2011	Risk of Falls	-0,045	0,420	-0,867	0,777	0,915	11	10
Community	Gschwind et al, 2015 KIN	Risk of Falls	0,153	0,262	-0,360	0,665	0,560	29	28
Community	Gschwind et al, 2015 SMT	Risk of Falls	0,055	0,208	-0,352	0,463	0,790	47	44
Community	Hsieh et al, 2014	Risk of Falls	-0,192	0,617	-1,401	1,017	0,756	4	4
Community	Lai et al, 2013a	Risk of Falls	0,279	0,357	-0,421	0,979	0,435	15	15
Community	Phirom et al, 2020c	Risk of Falls	0,830	0,328	0,188	1,472	0,011	19	20
Community	Pina et al, 2015	Risk of Falls	0,197	0,474	-0,732	1,126	0,678	8	8
Community	Rendon et al, 2012	Risk of Falls	0,020	0,335	-0,637	0,678	0,952	16	18
Community	Sato et al, 2015	Risk of Falls	0,266	0,263	-0,249	0,780	0,311	29	28
Community	Szturm et al, 2011	Risk of Falls	1,366	0,417	0,548	2,183	0,001	13	14
Community	Treml et al, 2013	Risk of Falls	0,109	0,345	-0,567	0,785	0,752	16	16
Community	Yang et al, 2020a	Risk of Falls	0,000	0,428	-0,839	0,839	1,000	10	10
Community	Yesilyaprak et al, 2016	Risk of Falls	0,107	0,461	-0,796	1,010	0,817	7	11
Community	Zahedian-Nasab et al, 2021	Risk of Falls	0,016	0,255	-0,483	0,516	0,949	30	30
Community			0,108	0,055	0,001	0,215	0,049		
Nursing Homes	Bieryla, Dold, 2014a	Risk of Falls	0,133	0,572	-0,988	1,254	0,816	5	5
Nursing Homes	Fu et al (2015)	Risk of Falls	0,804	0,265	0,284	1,324	0,002	30	30
Nursing Homes	Mugueta-Aguinaga; Garcia-Zapirain, 2017	Risk of Falls	1,127	0,339	0,463	1,791	0,001	20	19
Nursing Homes	Yesilyaprak et al, 2016a	Risk of Falls	0,424	0,466	-0,489	1,337	0,362	7	11
Nursing Homes	Zahedian-Nasab et al, 2021a	Risk of Falls	0,506	0,259	-0,002	1,014	0,051	30	30
Nursing Homes			0,685	0,148	0,394	0,975	0,000		
Overall			0,177	0,051	0,076	0,278	0,001		

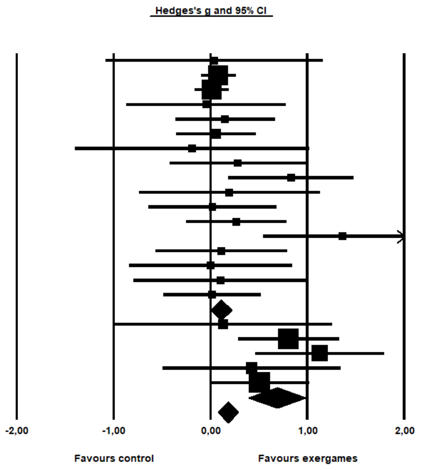

Place	I^2	P
Community Centers	3,491%	0,413
Nursing homes	0%	0,459

There was significance in both subgroups. The summary measure, which combines the subgroups, remains significant.

There was heterogeneity between the subgroups (P < 0,001), but not within subgroups (P = 0.44). Therefore, these subgroups more effectively explained the heterogeneity among studies.

6.2 Balance

Figure 5: Results of all measurements – balance studies

Study name	Outcome	Hedges'g	Lower limit	Upper limit	p-Value	Exergame	Control
		Statistics for each study				Sample size	
Bateni, 2012	Balance	-0,479	-1,582	0,624	0,395	5	6
Bieryla, Dold, 2014a	Balance	0,000	-1,120	1,120	1,000	5	5
Bieryla, Dold, 2014b	Balance	-0,061	-1,181	1,059	0,915	5	5
Cho et al, 2013a	Balance	1,797	0,990	2,605	0,000	17	15
Cho et al, 2013b	Balance	1,979	1,147	2,812	0,000	17	15
Delbaere et al, 2021	Balance	0,098	-0,077	0,272	0,273	254	249
Franco et al, 2011	Balance	0,524	-0,313	1,361	0,220	11	10
Hsieh et al, 2014	Balance	1,101	-0,219	2,422	0,102	4	4
Lai et al, 2013a	Balance	0,413	-0,291	1,117	0,250	15	15
Lai et al, 2013b	Balance	0,514	-0,194	1,223	0,155	15	15
Lai et al, 2013c	Balance	0,842	0,113	1,570	0,024	15	15
Lai et al, 2013d	Balance	0,151	-0,546	0,849	0,671	15	15
Lai et al, 2013e	Balance	0,380	-0,323	1,083	0,290	15	15
Lai et al, 2013f	Balance	0,401	-0,303	1,105	0,264	15	15
Pina et al, 2015	Balance	-0,371	-1,306	0,565	0,437	8	8
Rendon et al, 2012	Balance	0,232	-0,428	0,892	0,491	16	18
Sato et al, 2015a	Balance	0,012	-0,500	0,525	0,962	29	28
Sato et al, 2015b	Balance	1,004	0,459	1,548	0,000	29	28
Szturm et al, 2011a	Balance	2,419	1,443	3,395	0,000	13	14
Szturm et al, 2011b	Balance	0,413	-0,327	1,153	0,274	13	14
Szturm et al, 2011c	Balance	1,134	0,342	1,926	0,005	13	14
Treml et al, 2013a	Balance	0,185	-0,492	0,862	0,593	16	16
Treml et al, 2013b	Balance	0,109	-0,567	0,785	0,752	16	16
Treml et al, 2013c	Balance	0,705	0,008	1,403	0,047	16	16
Treml et al, 2013d	Balance	1,022	0,302	1,743	0,005	16	16
Treml et al, 2013e	Balance	0,000	-0,675	0,675	1,000	16	16
Yang et al, 2020a	Balance	-0,001	-0,841	0,838	0,997	10	10
Yang et al, 2020b	Balance	0,726	-0,143	1,596	0,101	10	10
Yang et al, 2020c	Balance	0,018	-0,821	0,858	0,966	10	10
Yesilyaprak et al, 2016a	Balance	0,307	-0,601	1,215	0,507	7	11
Yesilyaprak et al, 2016b	Balance	0,255	-0,651	1,162	0,581	7	11
Yesilyaprak et al, 2016c	Balance	-0,284	-1,191	0,623	0,540	7	11
Yesilyaprak et al, 2016d	Balance	1,179	0,198	2,161	0,018	7	11
Yesilyaprak et al, 2016e	Balance	0,762	-0,175	1,698	0,111	7	11
Yesilyaprak et al, 2016f	Balance	-0,051	-0,953	0,852	0,912	7	11
Yesilyaprak et al, 2016g	Balance	1,059	0,092	2,025	0,032	7	11
Zahedian-Nasab et al, 2021	Balance	0,420	-0,085	0,925	0,103	30	30

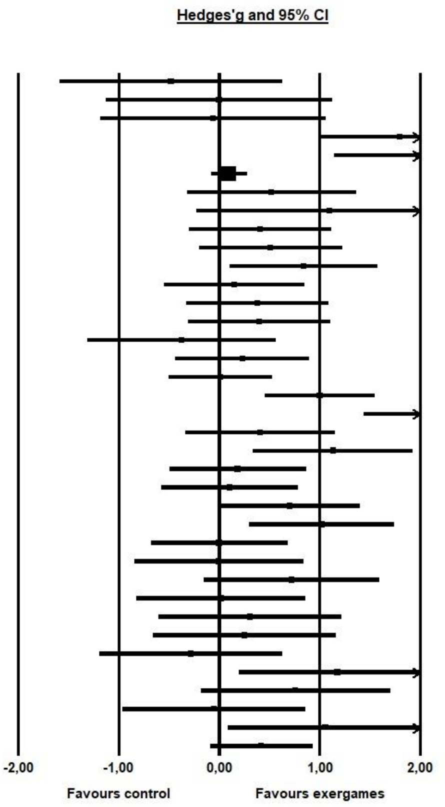

Figure 6: Subgroup analysis: organization by the control group - with or without intervention (balance studies)

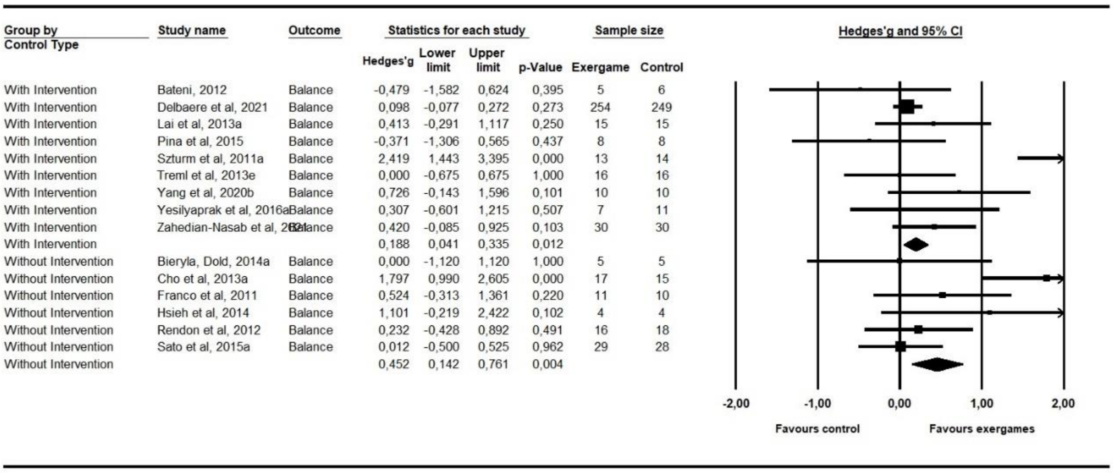

Interventions	I^2	p
With Interventions	70.291%	0.007
Without Interventions	67.757%	0.008

There was heterogeneity within groups (P < 0.001) but not between groups (P = 0.132). Both summary measures were significant. There were great and significant heterogeneities.

Subgroup analysis by duration of intervention was not performed, as there were only two studies in the group of studies with 12 weeks or more.

Figure 7: Subgroup analysis – place of execution (balance studies)

Group by Place	Study name	Outcome	Statistics for each study				
			Hedges's g	Standard error	Lower limit	Upper limit	p-Value
Community	Cho et al, 2013a	Balance	1,797	0,412	0,990	2,605	0,000
Community	Delbaere et al, 2021	Balance	0,098	0,089	-0,077	0,272	0,273
Community	Franco et al, 2011	Balance	0,524	0,427	-0,313	1,361	0,220
Community	Hsieh et al, 2014	Balance	1,101	0,674	-0,219	2,422	0,102
Community	Lai et al, 2013a	Balance	0,413	0,359	-0,291	1,117	0,250
Community	Pina et al, 2015	Balance	-0,371	0,477	-1,306	0,565	0,437
Community	Rendon et al, 2012	Balance	0,232	0,337	-0,428	0,892	0,491
Community	Sato et al, 2015a	Balance	0,012	0,261	-0,500	0,525	0,962
Community	Szturm et al, 2011a	Balance	2,419	0,498	1,443	3,395	0,000
Community	Szturm et al, 2011	Balance	1,134	0,404	0,342	1,926	0,005
Community	Treml et al, 2013e	Balance	0,000	0,345	-0,675	0,675	1,000
Community	Yang et al, 2020b	Balance	0,726	0,443	-0,143	1,596	0,101
Community			0,600	0,197	0,215	0,986	0,002
Nursing Homes	Bateni, 2012	Balance	-0,479	0,563	-1,582	0,624	0,395
Nursing Homes	Bieryla, Dold, 2014a	Balance	0,000	0,571	-1,120	1,120	1,000
Nursing Homes	Yesilyaprak et al, 2016a	Balance	0,307	0,463	-0,601	1,215	0,507
Nursing Homes	Zahedian-Nasab et al, 2021	Balance	0,420	0,258	-0,085	0,925	0,103
Nursing Homes			0,241	0,196	-0,144	0,626	0,220
Overall			0,420	0,139	0,148	0,693	0,002

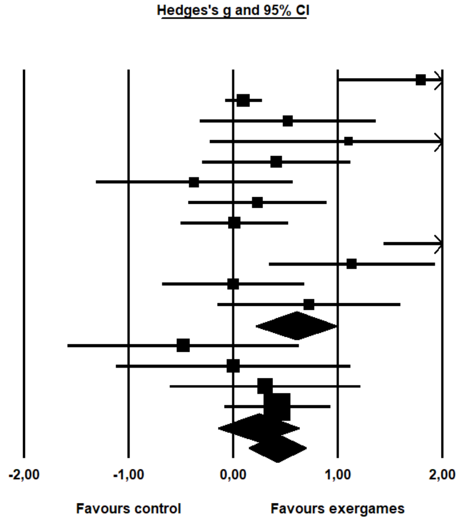

Place	I^2	P
Community Centers	76,685%	<0,001
Nursing homes	0%	0,509

There was heterogeneity within groups (P < 0.001) but not between groups (P = 0.914). There were great and significant heterogeneities only in the “community centers” group.

There was significance only in the “community centers” group. The summary measure that combines the subgroups remains significant.
