# Supplementary material for: Exergames in Older Adult Community Centers and Nursing Homes to Improve Balance and Minimize the Risk of Falls in Older Adults: A Systematic Review and Meta-Analysis
Source: Healthcare (Basel). 2023 Jun 28;11(13):1872. doi: 10.3390/healthcare11131872 (PMC10341223; doi:10.3390/healthcare11131872)
Supplement: Supplementary file 1 [file healthcare-11-01872-s001.zip › Supplementary Material S4 - updated.pdf]

Supplementary material 4: Overview of the main characteristics of the studies retrieved on the systematic review

Study	Sample	Study design	Adherence	Measured Outcomes	Results and Conclusions
(Fu , Gao, Tung, Tsang, & Kwan, 2015) [57]	***N:*** 60 older adults<br><br>***Age (means):*** Intervention group: 82.4 (3.8) years Control group: 82.3 (4.3) years<br><br>***Characteristics:*** All participants lived in a nursing home.<br><br>***Residential Location:*** Nursing Homes	***Duration:*** 6 weeks (18 sessions, 3 times for week, plus 12 months of surveillance)<br><br>***Intervention:*** Experimental Group participated in balance games and Control Group in Conventional Balance Training<br><br>***Technology Used:*** Nintendo Wii Fit Balance<br><br>***Research Design:*** Randomized Clinical Trial (RCT)	***Adherence:*** **"**All 60 participants completed the 6-week training program and the postintervention assessment. Two participants from the Wii Fit balance training group and 3 from the conventional balance training group could not complete the full year of surveillance because of illness or death, so the completion rates were 93.3% for the Wii Fit balance training group and 90% for the conventional balance training group."	***Risk of falls:*** PPA<br><br>***Balance:*** Not applicable	***Results:*** Physiological Profile Assessment scores and the incidence of falls significantly improved in both groups after intervention, but participants in the Wii Fit training group showed significantly greater improvement on both measures.<br><br>**Authors' conclusions:** In institutionalized elderly people with a history of falls, Wii Fit balance training was more effective than conventional balance training in reducing the risk and incidence of falls.
(Szturm, Betker, Moussavi, Desai, & Goodman, 2011) [58]	***N:*** 30 older adults<br><br>***Age (median):*** Intervention group – 80.5 years Control group – 81 years<br><br>***Characteristics:*** Community elderly who	***Duration:*** 8 weeks (16 sessions, 2 times for week)<br><br>***Intervention:*** Experimental Group participated in a dynamic balance exercise program coupled with the video game. The Control Group participated in a typical rehabilitation	***Adherence:*** *Intervention group:* **"**Completed all sessions (n=13). Did not complete all sessions (n=1 opted to not continue with program; n=1 due to medical reasons)." Membership fee: 86,67%<br><br>*Control Group:* **"**Completed all sessions (n=14). Did not complete all sessions (n=1 opted to not continue with	***Risk of falls:*** TUG<br><br>***Balance:*** BBS ABC LOB	***Results:*** The results showed significant improvements in post-treatment balance for both groups, and a significantly greater improvement was observed in the experimental group compared to the control group, except in the Time Up and Go test or space-variation test. brand temporal, for both groups.<br><br>**Authors' conclusions:** Dynamic balance exercises in video games were considered feasible. The improvement in balance was greater in the exergame group, but there was no difference in the gait function test.

	attended a day hospital.<br><br>***Residential Location:***<br>Community	program consisting of strengthening and balance exercises.<br><br>***Technology Used:***<br>Computer Game<br><br>***Research Design:***<br>Randomized Clinical Trial (RCT)	assessment)" Membership fee: 93,33%		
(Batista, Wibelinger, De Marchi, & Pasqualotti, 2014) [51]	***N:*** 38 older adults (Woman)<br><br>***Age (means):***<br>68.1 (6.2) years (did not specify by group).<br><br>***Characteristics:***<br>Elderly women had balance problems.<br><br>***Residential Location:***<br>Community	***Duration:*** 5 weeks (20 sessions, 2 times for week)<br><br>***Intervention:***<br>Participated in a balance intervention program, through six games on the Exergame platform.<br><br>***Technology Used:***<br>Nintendo Wii Fit Balance<br><br>***Research Design:***<br>Quasi-experimental study	***Adherence:***<br>All participants completed the intervention<br>Membership fee: 100.0%	***Risk of falls:***<br>Not applicable<br><br>***Balance:***<br>BBS<br>Center of gravity	**Results**: In the present study, there was a statistically significant improvement in the balance of the elderly when compared before and after the intervention, in addition, the six games played also showed statistical significance when comparing the first and twentieth sessions, highlighting the games Tightrope Walk, Table Tilt, Deep Breathing and Soccer Heading.<br><br>**Authors' conclusions:** Balance assessment and training with the Wii Balance Board platform were able to provide significant results for the elderly, in addition, the video game has the potential to treat the health, well-being and functional capacity of the elderly through real-time visual representation of the game.
(Pina et al., 2015) [52]	***N:*** 16 older adults<br><br>***Age (means):***<br>Intervention group: 70.2 (7.32) years<br>Control group: 67.2 (5.52) years	***Duration:*** 8 weeks (24 sessions, 3 times for week)<br><br>***Intervention:*** The Experimental group participated in an aerobic exercise program with the video game. The control group	***Adherence:***<br>Intervention group: 4 dropouts due to more than 2 absences per week. Membership fee: 50%<br><br>Control group: 3 dropouts due to knee joint pain.<br>Membership fee: 62.5%	***Risk of falls:***<br>TUG<br><br>***Balance:***<br>BBS	***Results:*** Both groups showed significant improvements in BBS and TUG. There were no significant differences between groups, that is, both resources were equally effective.<br><br>**Authors' conclusions:**Both the stationary bicycle exercises and the exergame are aerobic training options to improve the balance and risk of falls in sedentary elderly people.

	Characteristics: Elderly people able to walk independently, without cognitive impairment and who had participated in a physical activity program in the last 6 months<br><br>***Residential Location:*** Community	participated in a continuous aerobic training program on an exercise bike. The intensity of both interventions was established with a value of 12-13 points on the Borg Scale.<br><br>***Technology Used:*** Nintendo Wii Fit Balance<br><br>***Research Design:*** Randomized Clinical Trial (RCT)			
(Cho, Hwangbo, & Shin, 2014) [59]	***N:*** 32 older adults<br><br>***Age (means):*** Intervention group – 73.1 (1.1) years Control group – 71.7 (1.2) years<br><br>***Characteristics:*** Elderly without cognitive impairment, without vision and hearing problems, with a MiniMental score of at least 23 and without a	***Duration:*** 8 weeks (24 sessions, 3 times for week)<br><br>***Intervention:*** The Exergame group participated in a balance game program. The control group received no intervention.<br><br>***Technology Used:*** Nintendo Wii Fit Balance<br><br>***Research Design:*** Randomized Clinical Trial (RCT)	***Adherence:*** Not informed, but from the results presented, it seems that there were no losses.	***Risk of falls:*** Not applicable<br><br>***Balance:*** Romberg Eyes Open Romberg Eyes Closed	***Results:*** In the Exergame group, balance improved significantly after the intervention, both with eyes open and with eyes closed. Still, there was a significant difference between the groups after the intervention.<br><br>**Authors' conclusions:** Exergame is effective to improve the balance of healthy elderly people, being recommended as a form of exercise to prevent falls in elderly people.

	diagnosis of disease orthopedic and fractures in the last 6 months.<br><br>***Residential Location:*** Community				
(Sato, Kuroki, Saiki, & Nagatomi, 2015) [17]	***N:*** 57 older adults<br><br>***Age (means):*** Intervention group: 70.07 (5.35) years Control group: 68.5 (5.47) years<br><br>***Characteristics:*** Elderly with normal score on MiniMental and normal score on Motor Fitness Scale, indicating that the elderly did not have significant motor disability.<br><br>***Residential Location:*** Community	***Duration:*** 12 weeks (24 sessions, 2 times for week)<br><br>***Intervention:*** The intervention group participated in an exergame program with games of balance, motor coordination, gait and strength. The control group received no intervention.<br><br>***Technology Used:*** Microsoft Kinect<br><br>***Research Design:*** Randomized Clinical Trial (RCT)	***Adherence:*** Intervention group: 1 discontinued intervention illness. Membership fee: 96.55%<br><br>Control group: 2 declined post-test. Membership fee: 92.85%	***Risk of falls:*** CS-30 test<br><br>***Balance:*** BBS<br>FRT	***Results:*** Walking, muscle strength and motor coordination significantly improved in participants in the experimental group after the intervention. Also, the exergame group significantly improved compared to the control group in tests performed after the intervention.<br><br>**Authors' conclusions:**The exergame developed in this study was considered effective in improving walking, muscle strength and balance in elderly people.
(Treml et al., 2013)	***N:*** 32 older adults	***Duration:*** 5 weeks (10 sessions)	***Adherence:*** Uniformed	***Risk of falls:*** POMA	***Results:*** There was no significant improvement in the Berg Balance Scale (BBS) in the experimental group

[53]	***Age (means):*** Intervention group: 66.88 years Control group: 67.63 years<br><br>***Characteristics:*** Healthy elderly, walking independently, without neurological and cardiorespirator y alterations.<br><br>***Residential Location:*** Community	***Intervention:*** The Experimental group participated in a training program with proprioceptive training using an exergame. The control group participated in a conventional proprioceptive training program.<br><br>***Technology Used:*** Nintendo Wii Fit Balance<br><br>***Research Design:*** Quasi-experimental study		***Balance:*** BBS<br>Unipedal Stance<br>FRT	before and after the intervention, but a significant improvement was observed in the POMA scale, Unipodal balance and functional reach test anterior and lateral. In the control group, significant improvements were observed in the POMA scale and unipodal balance in the pre- and post-intervention moments.<br><br>**Authors' conclusions:**It is concluded that proprioceptive training with virtual reality proved to be more efficient than conventional proprioceptive training in elderly individuals in relation to balance, mobility, flexibility and falls.
(Bieryla and Dold, 2013) [12]	***N:*** 12 older adults<br><br>***Age (mean):*** 81.5 (5.5) years (did not specify by group)<br><br>***Characteristics:*** All seniors lived independently, were able to stand unaided for 30 minutes and walk for at least 6 meters without	***Duration:*** 3 weeks (9 sessions, 2 times for week)<br><br>***Intervention:*** The experimental group participated in exergame training through yoga games, aerobic and balance exercises. The control group continued with the conventional activities already practiced.<br><br>***Technology Used:*** Nintendo Wii Fit	***Adherence:*** Intervention group: 1 dropout after one week of intervention. 1 dropout per appointment after the end of the intervention. Membership fee: 66.66%<br><br>Control group: 1 dropout after one week of intervention. Membership fee: 83.33%	***Risk of falls:*** TUG<br>FAB<br><br>***Balance:*** BBS<br>FRT	***Results:*** The experimental group significantly increased their Berg Balance Scale (BBS) after training, while the control group did not. There was no significant change for any of the groups for the other tests analyzed.<br><br>**Authors' conclusions:**Exergame balance training may be a new way for seniors to improve balance measured by the BBS.

	assistance. Elderly people without neurological or vestibular disorders prior to the study.<br><br>***Residential Location:***<br>Nursing Homes	Balance<br><br>***Research Design:***<br>Randomized Clinical Trial (RCT)			
(Gschwind et al., 2015) [56]	***N:*** 220 older adults<br><br>***Age (means):***<br>Intervention group (KIN): 80.1 (6.3) years<br>Inervention group (SMT): 82.5 (7.0) years<br>Control group: 80.2 (6.5) years<br><br>***Characteristics:***<br>The elderly lived independently, able to walk with or without assistance, able to watch TV with or without glasses. Elderly people with color blindness, neurodegenerati	***Duration:*** 16 weeks (no more informations)<br><br>***Intervention:***<br>Participants were divided into three groups: two Exergame groups, the Step-Mat-Training (SMT) and Microsoft Kinect (KIN) and a control group. The control group received no intervention. Assessment measures included risk of falling, muscle strength, reaction time, proprioception, vision, balance.<br><br>***Technologies Used:***<br>Microsoft Kinect and Step-Mat-Training<br><br>***Research Design:***<br>Randomized Clinical	***Adherence:***<br>Intervention group (KIN): 57 were recruited. 29 were allocated to the intervention group. 24 participants completed the training period and were included in the analises. Membership fee: 42.1%<br><br>Intervencion group (SMT): 91 were recruited. 47 were allocated to the intervention group. 39 participants completed the training period and were included in the analises. Membership fee: 42.85%<br><br>Control group: 72 participants were recruited to the control group, divided into the KIN intervention control group (28) and SMT intervention control group (44). 61 participants were in	***Risk of falls:***<br>TUG<br>PPA<br><br>***Balance:***<br>Not applicable	***Results:*** Compared to the control group, SMT participants improved their risk of falling, proprioception, reaction time, sitting and standing performance. On the other hand, the KIN group improved, in relation to the control group, muscle strength and vision, in addition to a tendency towards an improvement in the risk of falling.<br><br>**Authors' conclusions:** The results suggest that it is feasible for the elderly to perform an unsupervised exercise program at home using exergames. Both interventions reduced the risk of falling. However, further refinement of systems is needed to improve adherence and maximize the benefits of exergames to provide fall prevention programs in nursing homes.

	ve, cardiovascular disease and psychiatric disorder were excluded.<br><br>***Residential Location:*** Community	Trial (RCT)	the control group at the end of the training period (KIN = 19; SMT = 42) and were included in the analyses. Membership fee: 84.72%.		
(Mugueta-Aguinaga and Garcia-Zapirain, 2017) [13]	***N:*** 40 older adults<br><br>***Age (mean):*** Intervention group: 85.47 (6.46) years Control group: 83.11 (9.01) years<br><br>***Characteristics:*** Elderly who did not perform programmed physical activity, elderly with a score greater than 90 on the Barthel index (measures the estimate of the degree of independence in activities of daily living).	***Duration:*** 3 weeks (9 sessions, 2 times for week)<br><br>***Intervention:*** The exergame group participated in an exercise program through the FRED game, developed by the researchers, to work on the risk of falls and frailty. The control group continued activities of daily living without scheduled physical activity.<br><br>***Technology Used:*** Microsoft Kinect<br><br>***Research Design:*** Randomized Clinical Trial (RCT)	***Adherence:*** Intervention group: no loss of follow-up<br><br>Control group: 1 lost to fallow-up (death). Membership fee: 95%	***Risk of falls:*** SPPB<br><br>***Balance:*** Not applicable	***Results:*** After the intervention, 60% of the elderly obtained a score of 10, which indicates a lower risk of evidencing frailty. The degree of compliance and adherence to the game was confirmed by 100% attendance to the sessions.<br><br>**Authors' conclusions:**The results support the hypothesis that FRED significantly reduced the presence and severity of the risk of falls and frailty in a sample of sedentary elderly, with the potential to modify the risk profile of the elderly.

	Residential Location: Nursing Homes				
(Rendon et al., 2012) [50]	***N:*** 40 older adults<br><br>***Age (mean):*** Intervention group> 85.7 (4.3) years Control group: 83.3 (6.2) years<br><br>***Characteristics:*** Seniors able to participate in physical activities for 45-60 minutes, with normal vision. Elderly people with orthopedic, neurological and circulatory problems were excluded.<br><br>***Residential Location:*** Community	***Duration:*** 6 weeks (18 sessions, 3 times for week)<br><br>***Intervention:*** The exergame group participated in a training program through games that worked on balance. The control group received no intervention.<br><br>***Technology Used:*** Nintendo Wii Fit<br><br>***Research Design:*** Randomized Clinical Trial (RCT)	***Adherence:*** Intervention group: "4 participants did not complete the study due to loss of interest, and/or arthritic discomfort." Membership fee: 80%<br><br>Control group: "2 of the 20 control participants did not show up to the post-test data collection." Membership fee: 90%	***Risk of falls:*** 8foot and Go<br><br>***Balance:*** ABC	***Results:*** Compared to the control group, the exergame group showed significant improvements for the Time Up and Go test and balance confidence in specific activities.<br><br>**Authors' conclusions:**exergames prove to be a useful tool to improve dynamic balance and confidence in the balance of elderly people.
(Franco, Jacobs, Inzerillo, & Kluzik, 2012) [49]	***N:*** 32 older adults<br><br>***Age:*** Intervention group: 79.8 (4.7) years	***Duration:*** 3 weeks (9 sessions, 2 times for week)<br><br>***Intervention:*** Participants were divided into three	***Adherence:*** "Thirty-eight residents were interested in participating and of these, 32 residents completed the study."<br><br>There is no record of the	***Risk of falls:*** Tinnet<br><br>***Balance:*** BBS	***Results:*** In the Berg Balance Scale (BBS), in the assessment of gait and balance and in the Tinetti balance test, there were no significant differences between groups. The exergame proved to be a fun form of exercise for seniors.<br><br>**Authors' conclusions:**The authors argue that although

	Matter of Balance group: 77.9 (6.9) years**<br>Control group: 76.9 (6.3) years<br><br>***Characteristics:*** Low-income seniors. To participate, the elderly had to live independently, be able to see TV clearly from 2.5 meters away, and be able to walk with or without assistance. Elderly individuals who were unable to stand up for at least 2 minutes, with reduced cognitive capacity, and incapable of engaging in physical activity for between 10 and 15 minutes were excluded.<br><br>***Residential Location:***	groups: an Exergame Wii Fit Group, with balance games in the exergame and complementary home exercises; a group of conventional balance exercises; a control group, which received no intervention.<br><br>***Technology Used:*** Nintendo Wii Fit Balance<br><br>***Research Design:*** Randomized Clinical Trial (RCT)	causes or groups of the two participants who dropped out.		they have not observed significant differences between groups, new studies with longer duration interventions may show better results.

	Community				
(Lai et al., 2013) [16]	***N:*** 30 older adults<br><br>***Age:***<br>Intervention group: 70.6 (3.5) years<br>Control group: 74.8 (4.7) years<br><br>***Characteristics:*** Were excluded with neurological problems such as Parkinson's disease, dementia, cerebral vascular acity; as well as elderly people with arthritis, visual impairment and cardiovascular disease that impair walking or elderly people unable to walk without assistance.<br><br>***Residential Location:*** Community	***Duration:*** 12 weeks (36 sessions, 3 times for week)<br><br>***Intervention:*** The elderly were divided into two groups. One group trained with exergame for six weeks and received no intervention for the next six weeks. The other group did not receive intervention with exergame for the first six weeks and received it for the next six weeks. The IVGB exergame consists of balancing games with visual sensory feedback, where seniors saw their performance on a computer screen.<br><br>***Technology Used:*** IUGB Stepping Exercise<br><br>***Research Design:*** Randomized Clinical Trial (RCT)	***Adherence:*** no loss of follow-up	***Risk of falls:***<br>TUG<br>FES<br><br>***Balance:***<br>BBS<br>UST<br>Bi Eyes Open AS<br>Bi Eyes Closed AS<br>Bi Eyes Open SV<br>Bi Eyes Closed SV	***Results:*** After the Exergame intervention, both groups showed improved balance based on the results of the following tests: the Berg Balance Scale (BBS), Modified Fall Effectiveness Scale (MFES), Timed Up and Go (TUG) and the Sway Velocity (SV) test. Also, the group that received the intervention within the first six weeks, retained performance for the next six weeks without intervention.<br><br>**Authors' conclusions:** Exergame training (IVGB) improves balance after the intervention and the benefits remain partially after the training is completed.

(Yeşilyaprak, Yildirim, Tomruk, Ertekin, & Algun, 2016) [60]	***N:*** 18 older adults<br><br>***Age (means):*** Intervention group: 70.1 (4.0) years Control group: 73.1 (4.5) years.<br><br>***Characteristics:*** Seniors should have a history of at least one fall in the last year, be able to walk at least 10 meters. Elderly people with impaired vision or hearing, score less than 21 on the MiniMental, with neurological diseases, were excluded.<br><br>***Residential Location:*** Nursing Homes	***Duration:*** 6 weeks (18 sessions, 3 times for week)<br><br>***Intervention:*** The experimental group (exergame) participated in a balance training program on the platform developed by the researchers. The control group participated in a conventional balance training program.<br><br>***Technology Used:*** BTS NIRVANA VR Interactiv System<br><br>***Research Design:*** Randomized Clinical Trial (RCT)	***Adherence:***<br>Intervencion group: 1 participants did not want to continue the study; 2 participants discontinued lack of time. Membership fee: 70%<br><br>Control group: no lost follow-up. Membership fee: 100%.	***Risk of falls:***<br>TUG<br>FES<br><br>***Balance:***<br>BBS<br>OLS Eyes Open Right foot<br>OLS Eyes Open Left foot<br>OLS Eyes Closed Right foot<br>OLS Eyes Closed Left foot<br>TS Eyes Open<br>TS Eyes Closed	***Results:*** Both groups significantly improved performance on the Berg Balance Scale (BBS), but the improvement was similar in both groups, indicating that neither method was superior.<br><br>**Authors' conclusions:** Similar improvements were found in balance and fall risk based on the exergame, with balance training and conventional balance training in older adults. Both training sessions were effective and the choice of method must be made by the professional, according to the patient's characteristics.
(Bateni, 2012) [48]	***N:*** 12 older adults<br><br>***Age (mean):*** Intervention group – 68 (8)	***Duration:*** 4 weeks (12 sessions, 3 times for week)<br><br>***Intervention:*** Division into three groups:	***Adherence:***<br>"One participant from the WI group (female, 91 years old) was considered to be an outlier. The improvement in her Berg Balance Scale score	***Risk of falls:***<br>Not applicable<br><br>***Balance:***<br>BBS	***Results:*** All participants, from all groups, improved their performance on the Berg Balance Scale (BBS) and Ball test scores. The PW group (also PT) performed better than the WI group on the BBS scale after treatment.

	years<br>Control group – 72 (12) years<br><br>***Characteristics:***<br>Elderly people who did not have physical therapy treatment, and who did not use computer games that could affect balance, and who have had two or more falls in the last year.<br><br>***Residential Location:***<br>Nursing Homes	Physiotherapy + Exergame (PW); Exergame (WI); Physiotherapy (PT). The exergame was used for training in balance games. Physiotherapy training consisted of standard training to increase strength, posture and balance.*<br><br>***Technology Used:***<br>Nintendo Wii Fit Balance<br><br>***Research Design:***<br>Randomized Clinical Trial (RCT)<br><br>** For this study, only the Exergame (WI) and Physiotherapy (PT) groups were considered. The results of the Physical Therapy + Exergame (PW) group were not used in this review.*	was more than five SDs from the mean improvement of the other participants (from 35 to 45). For this reason, her data were considered questionable and were excluded from the analysis. Due to the double-blind nature of this study, it was not possible to identify the exact reason for the significant changes in her performance."<br><br>**For this study, only the Exergame (WI) and Physiotherapy (PT) groups were considered. Results from the Physical Therapy + Exergame (PW) group were not used in this review.*		**Authors' conclusions:**Wii Fit training seems to improve balance. However, physical therapy training on its own or in addition to Wii Fit training appears to improve balance to a greater extent than training with Wii Fit alone.
(Hsieh et al., 2014) [54]	***N:*** 8 older adults<br><br>***Age (means):***<br>Intervention group: 60.25 years<br>Control group: 67.25 years<br><br>***Characteristics:***	***Duration:*** 6 weeks (30 sessions, 5 times for week)<br><br>***Intervention:*** The Experimental group performed a training program with Exergame for balance and risk of falls. The games were	***Adherence:***<br>No loss of follow-up	***Risk of falls:***<br>TUG<br><br>***Balance:***<br>BBS	***Results:***<br>In the two tests applied, the Berg Balance Scale (BBS) and Time Up and Go (TUG), the elderly significantly improved compared to the control group.<br><br>**Authors' conclusions:**<br>*based* on the results, the developed exergame is able to improve the balance and risk of falls among the elderly.

	Elderly of both sexes, without cognitive problems.<br><br>***Residential Location:***<br>Community	designed to work on balance, hand coordination and reaction time. The control group received no intervention.<br><br>***Technology Used:***<br>Microsoft Kinect<br><br>***Research Design:***<br>Clinical Trial (CT)			
(Delbaere et al, 2021) [19]	***N:***<br>503 older adults<br><br>***Age (mean):***<br>Intervencion group - 77.1 (5.5) years<br>Control group – 77.7 (5.5) years<br><br>***Residential Location:***<br>Community	***Duration:*** 9 weeks (18 sessions, 2 times for week, plus follow-up program and intervention for 12 or 24 months)<br><br>***Intervention:***<br>503 people aged 70 years and older who were independente in activities of daily living, without cognitive impairment, progressive neurological disease, or any other unstable or acute medical condition precluding exercise.<br><br>***Technology Used:*** The StandingTall Programe<br><br>***Research Design:***<br>Randomized Clinical Trial (RCT)	***Adherence:***<br>After 12 months (9 program-related losses; 1 allocation-related; 10 health-related losses; 9 no interest in the program). Membership fee: 8858%<br>After 24 months (5 related to the program; 9 related to health problems; 3 not interested in te program). Membership fee:81.89%	***Risk of falls:***<br>PPA<br>TUG<br>FES<br>Maximum Lean Rang AP<br>5 times sit to stand test<br>10m walk<br>Short Physical Performance battery<br><br>***Balance:***<br>Not applicable	***Results:***<br>The falls rates were not statistically different in the two groups after the first 12 months in the intervention group; in the control group. Additionally, the proportion of people who fell was not statistically different at 12 months. However, the intervention group had a 16% lower rate of falls over 24 months and a 20% lower rate of injurious falls over 24 months compared with the control group.<br><br>**Authors' conclusions:**<br>The Exergaming balance exercise did not significantly affect the primary outcomes of this study. However the programme significantly reduced the rate of falls in infurious falls over two years, with similar but not statistically significant effects at 12 months. The exergaming program cout provide promising scalable fall prevention strategies.

(Zahedian-Nasab et al ,2021) [61]	***N:***<br>60 older adults<br><br>***Age:***<br>Intervencion group – 69.67 (7.72)<br>Control group – 72 (7.81)<br><br>***Characteristics:***<br>This clinical trial was performed on 60 elderly individuals living in nursing homes divided into two groups of control and Xbox.<br><br>***Residential Location:***<br>Nursing Homes	***Duration:***<br>6 weeks (12 sessions, 2 times for week)<br><br>***Intervention:***<br>The participants in the intervention group received VR exercises based on Xbox Kinect in form of two 30–45-min sessions held on a weekly basis for 6 weeks. The individuals in the control group, on the other hand, received routine exercises of the nursing homes. The research tools used in this study included a demographic questionnaire, the Berg Balance Scale (BBS), the Timed Up and Go (TUG) test, and the Falling Efficacy Scale (FES).<br><br>***Technology Used:***<br>Microsoft Kinect<br><br>***Research Design:***<br>Randomized Clinical Trial (RCT)	***Adherence:***<br>No loss of follow-up<br><br>***Intervention:***<br>This clinical trial was performed on 60 elderly individuals living in nursing homes divided into two groups of control and Xbox. The participants in the intervention group received VR exercises based on Xbox Kinect in form of two 30–45-min sessions held on a weekly basis for 6 weeks. The individuals in the control group, on the other hand, received routine exercises of the nursing homes. The research tools used in this study included a demographic questionnaire, the Berg Balance Scale (BBS), the Timed Up and Go (TUG) test, and the Falling Efficacy Scale (FES).	***Risk of falls:***<br>TUG<br>FES<br><br>***Balance:***<br>BBS	***Results:***<br>The findings of the current study demonstrated that the scores of BBS and TUG test as the indices of balance among elderly people improved significantly in the Xbox group after the intervention ($p < 0.001$ for both BBS and TUG test). Moreover, the score of fear of falling diminished significantly in the intervention group compared to the control group ($p < 0.001$).<br><br>**Authors' conclusions:**<br>According to the results of the presente investigation, 6 weeks of VR balance exercises could enhance balance and fear of falling among elderly people living in nursing homes.
(Phirom et al, 2019) [42]	***N:***<br>40 older adults<br><br>***Age:***	***Duration:***<br>12 weeks (36 sessions, 3 times for week)	***Adherence:***<br>"Forty eligible participants were enrolled and randomized to either the	***Risk of falls:***<br>PPA<br>TUG	***Results:***<br>Thirty-nine participants (mean age = 69.81 _ 3.78 years) completed the study (97.5%). At the end of the trial, participants in the intervention group

	Intervention group – 70.21 (4.18)<br>Control group – 69.40 (3.38)<br><br>***Characteristics:***<br>This study investigated the efects of interactive physical-cognitive game-based training on the fall risk and cognitive performance of older adults.<br><br>***Residential Location:***<br>Community	***Intervention:***<br>Participants in the interventioon group practiced an interactive game-based training program, for 60 minuts per session, 3 sessions per week, for 12 consecutive weeks. Participants in the control group received educational material covering cognitive enhancement and fall prevention strategies.<br><br>***Technology Used:***<br>Microsoft Kinect<br><br>***Research Design:***<br>Randomized Clinical Trial (RCT)	intervention group (n = 20) or control group (n = 20). Thirty-nine participants completed the study, which corresponded to na overall drop-out rate of 2.5%. One participant in the training group could not attend the re-assessment, due to health reasons unrelated to the study. Participants in the intervention group attended on average 35.6 sessions (98.8%) and no adverse events were reported."	***Balance:***<br>Not applicable	demonstrated significant improvement in the PPA fall risk score (p = 0.015), postural sway (p = 0.005), MoCA score (p = 0.001), and TUG-dual task (p = 0.045) compared to controls.<br><br>**Authors' conclusions:**<br>The interactive physical-cognitive, game-based training was e_ective in reducing physiological fall risk and improving cognitive function in community-dwelling older adults.
(Yang et al, 2020) [55]	***N:***<br>20 older adults<br><br>***Age (median):***<br>Intervencion group – 68.71 (64.09 – 74.84)<br>Control group – 67.54 (62.08 – 76.75)<br><br>***Residential Location:***	***Duration:***<br>5 weeks (10 sessions, 2 times for week)<br><br>**Intervention:**<br>Participants in the experimental group had 14 exergame sessions, alternating dance, strength, and endurance games. The control group had 14 training sessions based on a	***Adherence:***<br>No loss of follow-up	***Risk of falls:***<br>TUG<br>30 seg CST<br><br>***Balance:***<br>FRT<br>OLST Eyes Open<br>OLST Eyes Closed	**Results:**<br>Within-group comparison between the pre-test and post-test indicated that significant differences existed in all of the 5 tests (30-sec CST, TUG, FRT, OLST with eyes open, and OLST with eyes closed) in the Kinect exercise group. To the Conventional exercise group, however, significant differences were only observed in 30-sec CST, FRT and OLST with eyes open. With regard to between-group comparison, significant differences were only found in FRT.<br><br>**Authors' conclusions:**<br>The findings of this study indicated that after 5 weeks

Community	course developed to prevent falls in elderly women. Both groups had training sessions lasting 45 minutes.<br><br>***Technology Used:***<br>Microsoft Kinect<br><br>***Research Design:***<br>Randomized Clinical Trial (RCT)		of intervention, the community older adults in both groups exhibited significant improvement in balance performance. While both interventions were effective, the Kinect exercise group demonstrated enhancement in more tests assessing balance ability, when compared with the conventional exercise group. A further probe into how Kinect exergames differed from conventional physical exercise revealed the significant effects of Kinect exergames on functional reach. Additional benefits observed in this study also suggested the use of Kinect exercise as a feasible, safe, and effective training method for improving community older adults' balance, promoting group cohesion, and increasing motivation to exercise.
